# Deep Brain Stimulation in the Nucleus Accumbens for Binge Eating Disorder: a Study in Rats

**DOI:** 10.1007/s11695-020-04697-9

**Published:** 2020-05-25

**Authors:** D. L. Marinus Oterdoom, Renske Lok, André P. van Beek, Wilfred F.A. den Dunnen, Marloes Emous, J. Marc C. van Dijk, Gertjan van Dijk

**Affiliations:** 1 Department of Neurosurgery, University of Groningen, University Medical Center Groningen, PO Box 30001, 9700 RB Groningen, the Netherlands; 2 Department of Behavioral Neuroscience, Groningen Institute for Evolutionary Life Sciences (GELIFES), University of Groningen, Nijenborgh 7, 9747 AG Groningen, the Netherlands; 3 Department of Endocrinology, University of Groningen, University Medical Center Groningen, PO Box 30001, 9700 RB Groningen, the Netherlands; 4 Department of Pathology and Medical Biology, University of Groningen, University Medical Center Groningen, Groningen, the Netherlands; 5 Department of Bariatric and Metabolic Surgery, Medical Center Leeuwarden, Leeuwarden, the Netherlands

**Keywords:** Deep brain stimulation, Nucleus accumbens, Binge eating disorder, Animal study

## Abstract

Binge eating disorder (BED), with its compulsive and addictive components, may often underlie weight regain after gastrointestinal bariatric surgeries. BED is therefore considered an exclusion criterion for these surgeries. Anecdotal reports suggest that deep brain stimulation (DBS) is an effective treatment for addictive disorders with, similar to BED, pathological changes in cerebral reward circuitry. We therefore assessed effect of DBS of the nucleus accumbens (NAC) in a rat model of BED. Twenty-one male obesity prone Wistar rats with DBS electrodes placed in NAC subregions were subjected to a binge eating protocol. Binge eating was significantly reduced with DBS during (NAC core) or before (NAC lateral shell) the binge. These outcomes provide a base to further explore the potential of DBS in the treatment of BED.

**Electronic supplementary material:**

The online version of this article (10.1007/s11695-020-04697-9) contains supplementary material, which is available to authorized users.

## Introduction

Binge eating disorder (BED) affects approximately 30% of obese patients. It is characterized by recurrent episodes of binge eating without compensatory behavior, e.g., vomiting or laxative abuse [1]. BED is associated with poor outcome after “gastrointestinal” bariatric surgery [2] and is therefore regarded as an exclusion criterion for these surgeries.

BED is hypothesized to result from maladaptive changes in the cerebral reward system. Neuro-anatomically, reward experience is mediated by a network of cortical regions and basal ganglia, with the nucleus accumbens (NAC) as a key structure in this circuitry. The NAC is implicated in mediating the reinforcing properties of food, sex, and drugs. In drug addiction, the NAC is crucial for incentive sensitization, in which particular cues can augment visual attraction and strengthen the desire for a substance. In BED, obesity develops similar to other addictive disorders, with food cues leading to eating [3].

In deep brain stimulation (DBS), the NAC is a recognized target for neuropsychiatric diseases. Clinical case reports and experimental animal data also indicate a therapeutic potential for DBS-NAC in obesity treatment [3]. Therefore, DBS-NAC was studied in a rat model of BED in order to substantiate target localization and to establish stimulation parameters, with the aim to support DBS-NAC as a novel treatment modality for BED in humans.

## Materials and Methods

See supplementary file 1 for details. Twenty-one outbred male diet-induced obesity prone Wistar rats (Harlan-NL) had bilateral DBS electrodes implanted in the NAC core (*n* = 7), NAC lateral shell (*n* = 7), or NAC medial shell (*n* = 7). They were subsequently subjected to a binge eating protocol. In short, this consisted of 2-h interruption of their regular low fat (LF) chow on weekdays, during which an empty food rack was provided during the antepenultimate hour of the light phase, immediately followed by 1-h access to a highly palatable high fat/high sucrose (HFS) diet. DBS started when binge intake stabilized after 3 weeks.

One-hour stimulation either started just before placement of the empty racks or just before provision of the HFS binge. Stimulation current (I) was 250 μA (or 125 μA, in case of locomotor side effects), pulse width (PW) was 60 μs, and stimulation frequency was 140, 50, and 10 Hz. After the study, electrode positions were histologically verified. Binge intake during DBS days was compared with baseline sessions on the day before and after stimulation. Based on planned comparison in which DBS was expected to reduce binge eating behavior, statistical significance was determined with a one-tailed Student’s *t* test.

## Results

**Fig. 1:**
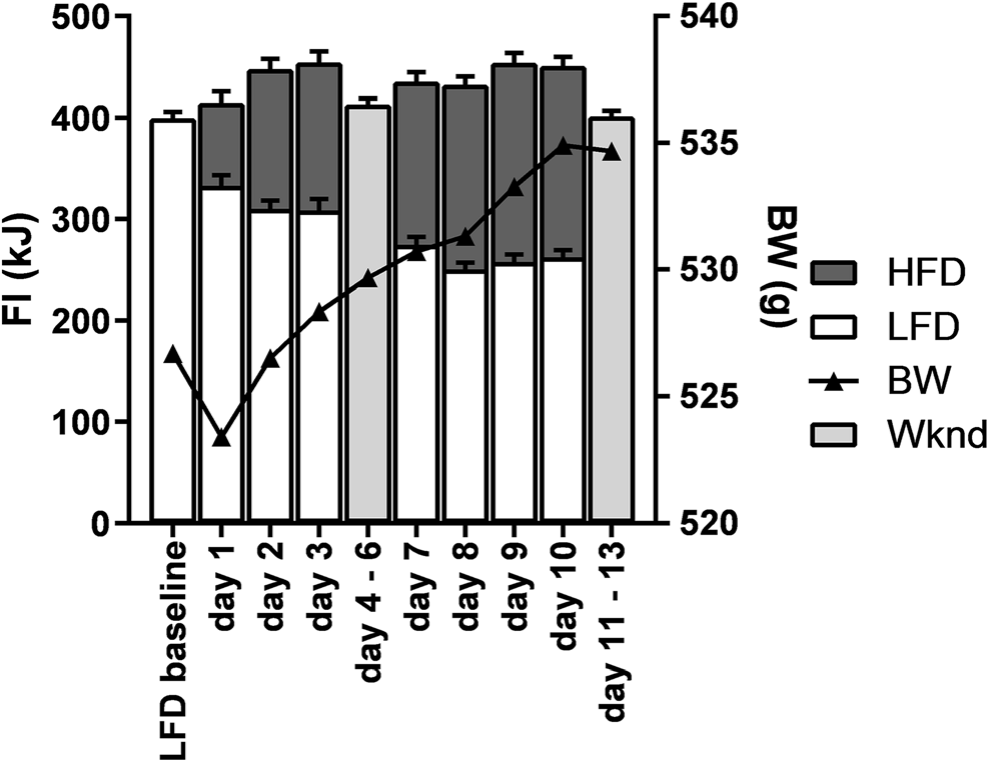
Daily intake of food during the 1 h access to the high fat/sucrose diet (HFS) and the 22 h access to the normal chow LF diet (which the rats also ate at baseline). During weekends, rats did not get access to the HFS binge but had continuous access to the LF diet

Daily caloric intake increased (*p* < 0.01) with the introduction of the binge protocol. HFS intake gradually increased at the expense of the LF intake, reaching a stable level of approximately 40% of total intake 2 weeks after introduction of the binge protocol. Total intake on binge days was higher compared with non-binge days in the weekends (Fig. 1). Body weight increased over the course of starting the binge protocol.

**Fig. 2:**
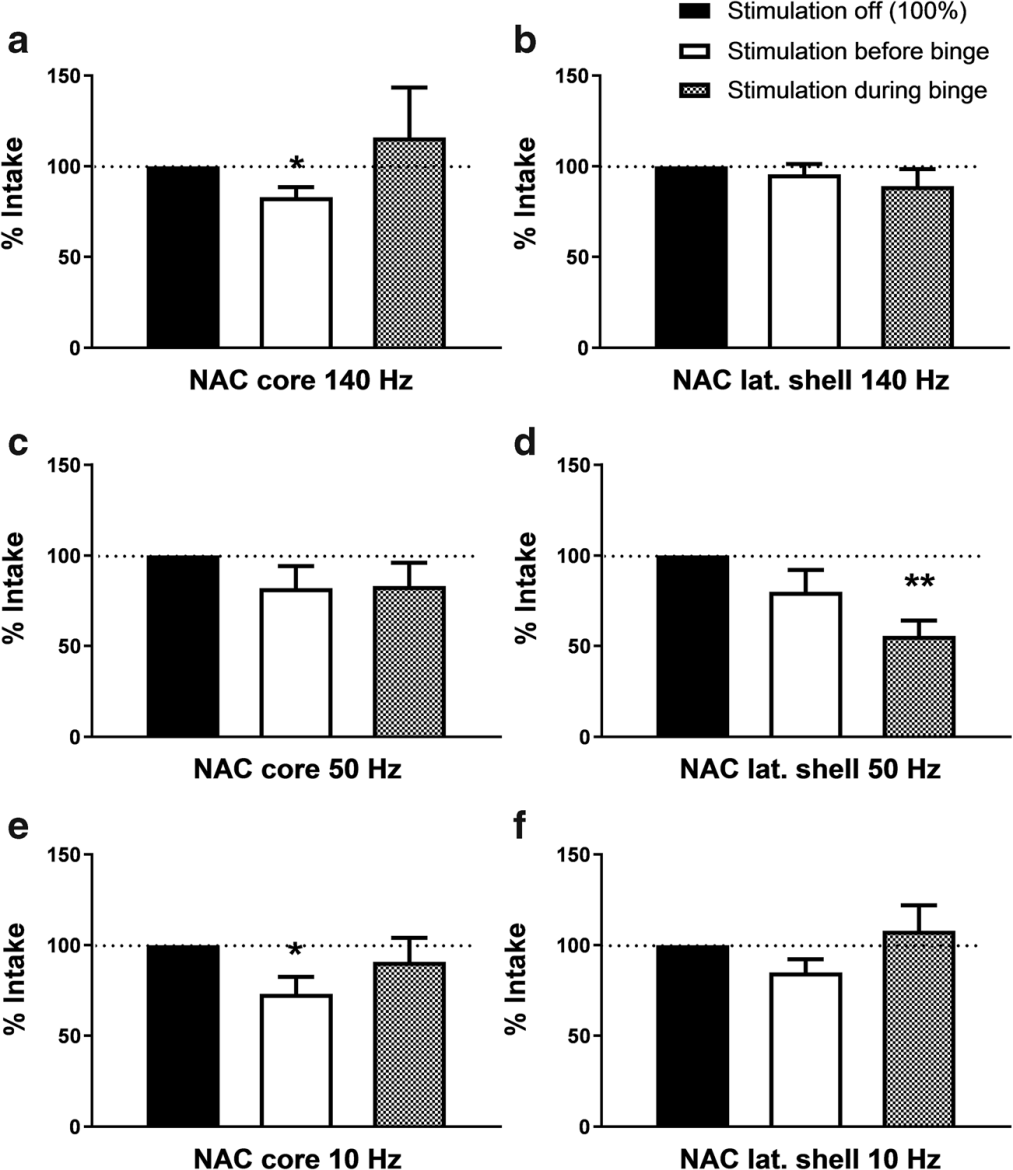
Deep brain stimulation in the nucleus accumbens core (NAC core, panels **a**, **c**, and **e**, with resp.140, 50, and 10 Hz) or lateral shell (NAC lat. shell, panels **b**, **d**, and **f**, with resp. 140, 50, and 10 Hz), in the hour before the binge (open bars) or during the binge (hatched bars). Relative to baseline intake (set at 100%, represented by the black bars), * denotes *p* < 0.05, ** denotes *p* < 0.01

High-frequency DBS (140 Hz) resulted in a significant decrease of binge intake when the NAC core was stimulated at 250 μA in the hour before the binge (intake 83 ± 5.6%, *p* = 0.02; 5 out of 6 rats eating less with stimulation). No effect was observed on binge intake when the NAC core was stimulated during the binge (Fig. 2, panel a). No significant decrease with high-frequency DBS in the NAC lateral shell (Fig. 2, panel b) or with lower current in the core (data not shown) were found. DBS of the NAC medial shell caused escape/fear behavior. Therefore, stimulation of the NAC medial shell was abandoned.

If rats showed aversive effects (i.e., excessive grooming behavior, head dips) when stimulating with a current of 250 μA/50 Hz, current was adjusted to 125 μA/50 Hz. A significant decrease in binge intake was found with DBS of the NAC lateral shell using adjusted current during the binge (intake 55.7 ± 8.4%, *p* = 0.003; all 6 rats eating less with stimulation) (Fig. 2, panel d). No significant decrease was found when stimulating the NAC lateral shell with 50 Hz before the binge (Fig. 2, panel d). No effect was found when stimulation the NAC core with 50 Hz either before or during the binge (Fig. 2, panel c). DBS did not alter the LF diet intake.

With low-frequency DBS (10 Hz), a significant decrease in binge intake was found when the NAC core was stimulated before the binge (intake 73.2 ± 9.3%, *p* = 0.03; 5 out of 6 rats eating less with stimulation, but not during the binge) (Fig. 2, panel e). Stimulation of the NAC lateral shell with 10 Hz did not alter binge intake, neither stimulating before nor during the binge (Fig. 2, panel f). None of the cases did DBS alter the LF diet intake.

## Discussion

In this study, the efficacy of DBS-NAC to reduce binge behavior was assessed in a rodent model of BED. The results show a decreased HFS intake with DBS of the NAC core *before* but not during the binge. In contrast, DBS of the NAC lateral shell led to suppression of HFS-intake *during* but not before the binge. These results indicate that DBS of the NAC core and lateral shell affect different aspects of binge eating behavior. DBS of the NAC medial shell was not tolerated, as it leads to induction of fear and escape behavior.

The dissociation of DBS effects in NAC core and lateral shell may be explained by the aspects of motivated behavior: “wanting” and “liking.” Wanting occurs in the anticipatory hour before the binge, when the empty rack is presented, and liking occurs during bingeing. Wanting is responsible for cravings, whereas liking is the pleasurable feeling of using the potentially incentive substance. Wanting and liking are mediated by dissociable brain systems [4]. Of note, rats in this study were pre-selected for a relatively high level of hyperphagia and weight gain upon 2-week ad libitum exposure to the HFS diet. In rats not pre-selected for HFS diet-induced obesity, DBS effects on binge intake were highly variable and did not yield significant results (data not presented).

These results can be explained by the work of Johnson and Kenny, who found addiction-like reward dysfunctions and compulsive eating related to the mesolimbic reward system in rats as a result of ad libitum exposure to a palatable HFS diet comparable to our study [5]. Besides alterations in mesolimbic circuitry resulting from ad libitum HFS exposure, the HFS diet-induced obesity selection procedure in our study may have preselected those rats that had (genetically) altered mesolimbic reward pathways to begin with.

### NAC Core—Wanting

In rodents, NAC neuronal firing rate is influenced by conditioned stimuli and feeding. Human neuroimaging studies show increased NAC activation in obese individuals in response to food stimuli [3]. This augmented “wanting” is thought to be reduced by DBS-NAC through the following mechanisms:

1. Hyperarousal of the mesolimbic system is caused by a shift in equilibrium between excitatory (glutamate) and inhibitory (GABA) neurotransmitters. Glutamate levels decrease and GABA levels increase with NAC core high-frequency DBS, which results in a decrease of addictive behavior [6].
2. High-frequency DBS of the NAC core inhibits release of dopamine by a decrease in spontaneous firing of dopaminergic neurons of the substantia nigra pars compacta (SNc) via a feedback loop from the striatum. Imbalance in the dopaminergic SNc-dorsal striatum projection system is thought to play a role in pathological habit formation, e.g., in obsessive compulsive disorder (OCD) [7]. By stimulating NAC core in the anticipatory phase, a decrease in dopamine release reduces craving and thus less eating.
3. Apart from the key role in dopaminergic signaling, the NAC is thought to act as a gateway. It selectively allows task-relevant inputs to pass to basal ganglia output regions and prevents task-irrelevant information from being processed by electrophysiological gating [8]. This gating might occur by cross-frequency coupling due to modulation of oscillatory activity in a frequency band according to the phase of another frequency band. In the reward circuitry, losing or winning money has been shown to elicit increased gamma oscillations (40–80 Hz) before and after decreased alpha oscillatory (8–12 Hz) activity. If electrophysiological gating is dysfunctional, irrelevant (food) cues can lead to pathologic behavior, e.g., overeating. DBS-NAC with 50 Hz activates gamma oscillations and may therefore restore dysfunctional electrophysiological gating [7].

### NAC Lateral Shell—Liking

Incentive hot spots and cold spots were identified in the NAC [9]. These hedonic hot spots were associated with “liking” operate via opioid receptors [10]. Micro-injections with μ-opioid receptor-stimulating substances cause doubling or tripling the positive hedonic response in reaction to sweet tastes. This makes food more “likeable.” It is hypothesized that DBS-NAC in the lateral shell results in less activation of hot spots, hence reducing the “liking” of addictive substances [8].

## Conclusion

This study demonstrates the beneficial effects of DBS-NAC on binge intake in a rodent model of BED. It is deducted that DBS intervenes on various, (potentially) dopamine-regulated behavioral aspects of motivated behavior. Frequency, current, and NAC sublocation have a major influence. These results are promising and encouraging, but further research is warranted to investigate the effects and adverse events of DBS-NAC in humans.

## Electronic supplementary material

ESM 1 (DOCX 18 kb)
