# Supplementary material for: Deep Brain Stimulation in the Nucleus Accumbens for Binge Eating Disorder: a Study in Rats
Source: Obes Surg. 2020 May 25;30(10):4145–8. doi: 10.1007/s11695-020-04697-9 (PMC7467950; doi:10.1007/s11695-020-04697-9)
Supplement: Supplementary file 1 — (DOCX 18 kb) [file 11695_2020_4697_MOESM1_ESM.docx]

**Animal Care and Surgery**

For the experimental procedures mentioned in this study, all applicable institutional and/or national guidelines for the care and use of animals were followed. Thirty male Wistar rats diet (Harlan-NL) were individually housed in controlled facilities (21⁰C, 50% humidity, 12h light-dark cycle) with free access to water and regular pelleted low fat (LF) chow containing 61.1 energy% carbohydrates, 13.9 energy% fat, and 25.0 energy% protein (RMHB – AB diets), and caloric content of 15.8 kJ. At the age of 6 months, LF diet was replaced for two weeks by a pelleted and highly palatable high fat/high sucrose diet (HFS) containing 31.7 energy% carbohydrates, 52.1energy% fat, and 16.39% protein and a caloric content of 21.1 kJoule. From this screening phase, 9 rats did not gain weight on the HFS diet and were considered anhedonic and excluded from further experiments. Then, while again on the LF diet, the 21 remaining rats underwent bilateral implantation of electrodes in the NAC core (n=7), NAC lateral shell (n=7), or NAC medial shell (n=7). Under isoflurane anesthesia, the head of the rats was placed in a stereotactic frame (Kopf, Germany). After subcutaneous lidocaine injection, a sagittal skin incision was made. Through a burr hole, gold plated electrodes were implanted [1]. Coordinates relative to the bregma:

• NACcore: medial-lateral (ml) 1.5 mm; dorsal-ventral (dv) 7.2 mm and anterior-posterior (ap) 1.6 mm

• NAC lateral shell: ml 2.5 mm; dv 8 mm and ap 1.6 mm.

• NAC medial Shell: ml 1 mm; dv 7.2 mm and ap 1.6 mm.

Electrodes were connected to a piece of IT circuit board, which then were individually connected with their inner and outer parts to one of 4 specific TE (male) connectivity pins that were adjusted on the other side of the circuitboard. A small hole inside the circuitboard was used for attachment to the stereotaxic arm used to place the electrodes. Once in place, the circuitboard was secured to the skull using dental cement anchored to 4 cranial screws placed in a square around the burr hole. Flunixine injection was postoperatively administered for pain relief. A protective socket was placed on the TE pins right after surgery, to prevent damage to the male TE pins. After regaining consciousness, rats were brought back to their vivarium, where they were daily checked.

**Binge protocol**

Three weeks after surgery, rats were habituated to the HFS binge protocol on weekdays. Food racks of rats containing the LF diet were replaced by empty food racks 3 hours before lights off. One hour later, a food rack containing the HFS diet was provided for 1 hour, after which it was replaced again by a food rack containing the LF diet. Empty food rack placement preceding the HFS exposure assured that rats experienced an anticipatory phase before the 1h-HFS binge started.

**DBS protocol**

Over the course of binge training, rats were subjected to daily connection to the stimulation wires on Tuesday till Friday. On Mondays, rats were not connected but were exposed to the binge protocol, in order to wash out the weekend effects of not binging. Rats were taken one by one from their home cage and connected to the wires in the hour before the empty food rack replaced the food rack with the LF diet. After assessing their body weight, the protective sockets on the connector pins placed were replaced by sockets connected to stimulation wires of approximately 1 meter long. These wires were connected to stimulation equipment outside the cage. A counterweight attached to the wires assured that the wires were not at reach for the rats.

DBS-experiments started as soon as baseline HFS-intake stabilized. A voltage-based stimulator (Grass SD9 EFS6K26SO) generated monophasic pulses. Constant current units (CCU1A Grass medical instruments) were used to convert tension into current and maintain stable current levels. Stimulus output was monitored using a calibrated oscilloscope (Tektronix, 2225, 50mHz). Currents were calibrated using a 10kΩ resistance. Animals were stimulated on two alternate days, separated from each other by at least a baseline session. Stimulation was started right before placement of the empty racks, or right before provision of the HFS binge. Stimulation parameters were based on literature studies [2,3,4]. Stimulation current (I) was 250 µA or 125 µA (in case of locomotor side effects occurred with 250 µA), pulse width (PW) was 60 µs, and stimulation frequency was tested at 140, 50 and 10 Hz.

**Electrode placement verification**

After the study, rats were anaesthetized with Na-pentobarbital, and transcardially perfused with heparinized saline as pre-rinse followed by 0.4% paraformaldehyde solution. Brains were removed and further fixed in phosphate-buffered 4% formalin ap pH 7.4. Electrodes were carefully extracted along their insertion shafts. Brain tissues were subsequently processed in a graded ethanol series followed by xylene and paraffination. Serial sections were cut, and stained with Haematoxilin&Eosin and Sevier-Munger silver stain. Deepest track-point of the electrode was compared with the stereotactic atlas of the rat brain. In each group, one rat was removed because of inappropriate electrode placement.

**Data analysis**

Total food-intake during DBS was compared to baseline sessions on the day before and after stimulation. Two baseline sessions were calculated as an average. A comparison was made between: (1) average baseline vs. stimulation during binge (2), average baseline vs. stimulation before binge. Based on planned comparison of which DBS was expected to reduce binge eating behavior, statistical significance was determined using a one-tailed Student’s t-test, with level of significance set at p<0.1.

**References**

1. Temel Y. Deep Brain Stimulation in Animal Models. Handb Clin Neurol. 2013;116:19-25.

2. Voges J, Müller U, Bogerts B et al. Deep brain stimulation surgery for alcohol addiction. World Neurosurg. 2010;80:28.e21-28.e31.

3. van der Plasse G, Schrama R, van Seters SP et al. Deep brain stimulation reveals a dissociation of consummatory and motivated behaviour in the medial and lateral nucleus accumbens shell of the rat. PLoS One. 2012;7:e33455.

4. Volkmann J, Herzog J, Kopper F et al, Introduction to the programming of deep brain stimulators. Mov Disord. 2002;17:181-87.
